# Cost of Illness Due to Severe Enteric Fever in India

**DOI:** 10.1093/infdis/jiab282

**Published:** 2021-11-23

**Authors:** Dilesh Kumar, Atul Sharma, Saroj Kumar Rana, Shankar Prinja, Karthikeyan Ramanujam, Arun S Karthikeyan, Reshma Raju, Swathi Krishna Njarekkattuvalappil, Prasanna S Premkumar, Akashdeep Singh Chauhan, Venkata Raghava Mohan, Sheena Evelyn Ebenezer, Mathew Santosh Thomas, Madhu Gupta, Ashita Singh, Dasaratha Ramaiah Jinka, Shajin Thankaraj, Roshine Mary Koshy, Christina Dhas Sankhro, Arti Kapil, Jayanthi Shastri, Karnika Saigal, Sulochana Putli Bai Perumal, Savitha Nagaraj, Shalini Anandan, Maria Thomas, Pallab Ray, Jacob John, Gagandeep Kang

**Affiliations:** 1 Christian Medical College, Vellore,India; 2 Postgraduate Institute of Medical Education and Research, Chandigarh,India; 3 The Duncan Hospital, Raxaul, Bihar,India; 4 Chinchpada Christian Hospital, Maharashtra,India; 5 Rural Development Trust Hospital, Bathalapalli, Andhra Pradesh,India; 6 Makunda Christian Leprosy and General Hospital, Bazaricherra, Assam,India; 7 Lady Willingdon Hospital, Manali, Himachal Pradesh,India; 8 All India Institute of Medical Sciences, New Delhi,India; 9 Topiwala National Medical College and BYL Nair Charitable Hospital, Mumbai,India; 10 Chacha Nehru Bal Chikitsalaya, New Delhi,India; 11 Kanchi Kamakoti Childs Trust Hospital, Chennai,India; 12 St John’s Medical College, Bangalore,India; 13 Christian Medical College and Hospital, Ludhiana,India

**Keywords:** cost of illness, economic burden, enteric fever, health expenditure, ileal perforation, India, out of pocket expenditure, typhoid

## Abstract

**Background:**

Lack of robust data on economic burden due to enteric fever in India has made decision making on typhoid vaccination a challenge. Surveillance for Enteric Fever network was established to address gaps in typhoid disease and economic burden.

**Methods:**

Patients hospitalized with blood culture-confirmed enteric fever and nontraumatic ileal perforation were identified at 14 hospitals. These sites represent urban referral hospitals (tier 3) and smaller hospitals in urban slums, remote rural, and tribal settings (tier 2). Cost of illness and productivity loss data from onset to 28 days after discharge from hospital were collected using a structured questionnaire. The direct and indirect costs of an illness episode were analyzed by type of setting.

**Results:**

In total, 274 patients from tier 2 surveillance, 891 patients from tier 3 surveillance, and 110 ileal perforation patients provided the cost of illness data. The mean direct cost of severe enteric fever was US$119.1 (95% confidence interval [CI], US$85.8–152.4) in tier 2 and US$405.7 (95% CI, 366.9–444.4) in tier 3; 16.9% of patients in tier 3 experienced catastrophic expenditure.

**Conclusions:**

The cost of treating enteric fever is considerable and likely to increase with emerging antimicrobial resistance. Equitable preventive strategies are urgently needed.

Enteric fever is a public health concern in many low and middle-income countries (LMICs). In 2017, 14.3 million enteric fever episodes resulted in an estimated 178 000 deaths globally, with 70% in South Asia alone. Enteric fever was also the cause of 6 737 500 years of life lost in South Asia [1]. With the emergence of antimicrobial resistance and slow progress on water and sanitation interventions, the burden of enteric fever may increase [2, 3].

Typhoid vaccination could provide control of the disease until water, sanitation, and hygiene interventions reap dividends [4]. The World Health Organization (WHO) recommended introducing typhoid conjugate vaccine (TCV) in LMIC countries [5]. However, this requires substantial investment for these economies. With only 1.15% of gross domestic product public spending towards health care, India’s vaccination program has been slow to introduce new vaccines and relies heavily on out-of-pocket payments for treatment [6]. As India transitions out of Gavi Alliance support, a policy decision on introducing a new vaccine is urgent and necessitates substantial epidemiological and economic evidence of enteric fever burden [7–9].

Disease-specific spending in India is not well documented. There are very few data sources of individual health care expenditure, with only 3 studies reporting typhoid fever costs [10]. The Diseases of the Most Impoverished (DOMI) program reported that the total cost incurred by a hospitalized case to be US$129 [11]. In Kolkata, the average treatment cost of a hospitalized patient was US$99.36 in 2 hospitals [12]. In an urban slum in New Delhi, the mean total cost (patient and provider) of typhoid fever was US$126, with a hospitalized case costing much higher, US$636 [13]. Pooling these data has been challenging due to the different settings, health care facilities, and methodologies. Hence there was a need to assess the out-of-pocket burden of patients suffering from enteric fever in rural and urban areas.

The Surveillance for Enteric Fever in India (SEFI) network was established to obtain reliable contemporaneous epidemiological data on enteric fever [14–16]. This article presents the out-of-pocket costs incurred by patients hospitalized with severe enteric fever and nontraumatic ileal perforation, a common complication of enteric fever [17], from the SEFI surveillance.

## METHODS

### Study Design and Setting

The SEFI network conducted surveillance at 18 sites across India for 2 years between November 2017 and March 2020. In tier 1 surveillance (4 sites), a large cohort of children younger than 15 years were followed for 24 months to measure the community incidence of typhoid fever. However, because the study supported the diagnostics and management of illness, tier 1 was not included in the cost estimation. The tier 2 surveillance was done in smaller hospitals in 5 rural sites and 1 urban site, combining facility-based surveillance with a health care utilization survey to estimate the incidence of severe enteric fever. The tier 3 surveillance was done in 8 key tertiary care hospitals in India. All blood culture-confirmed enteric fever patients in these hospitals were enrolled to assess patterns of enteric fever caused by *Salmonella* Typhi and *Salmonella* Paratyphi and estimate their antimicrobial resistance [14]. The 14 hospitals in tiers 2 and 3 captured cost of illness data for this study.

### Data Collection

Severe enteric fever was defined as a hospitalized case with blood culture-confirmation for enteric fever. Nontraumatic ileal perforation was defined as a case diagnosed with ileal perforation by the operating surgeon.

The economic data was obtained from the hybrid and laboratory surveillance using an incidence-based approach with an additional recall component for collecting pre- and posthospitalization costs using a structured questionnaire [18]. It included sociodemographic characteristics, income-expenditure details, illness-specific expenses, productive time lost, alternatives used for productivity, and related costs. Patients were followed up until 28 days to understand the costs incurred for the entire episode of illness. All collected information was entered electronically into a cloud-based server via a secure data management system. Data validation was done at the point of entry with inbuilt range and internal consistency checks. Simultaneously, the data management team performed standard logic checks and resolved issues using a closed query redressal system.

### Data Analysis

Data analysis was done with Stata 15.1 software package. Incomplete data were excluded from the final analysis. All cost-related information was reported in Indian Rupees (Rs) and US dollars, wherein 2 January 2019 (mid-year of surveillance) currency conversion rate (1 US$ = Rs 69.6089) was applied [19].

The direct cost of 1 episode of severe enteric fever included hospitalization charges and outpatient charges pre- and posthospitalization. It comprises medical costs, including bed/consultation, diagnostic tests, procedure costs, prescribed medicines, and nonmedical costs such as food, transport, lodging, other expenses, and productive time lost by patients and caregivers. Indirect costs were computed from the patient and caregiver’s income loss and costs related to alternative productivity arrangements. The effective income loss was computed, assuming the patient or caregiver would work an 8-hour shift for 26 days a month. The total indirect cost was the sum of income lost and the payments related to alternate productivity used. Income lost was equal to the total time lost in professional work (hours) multiplied by the gross hourly income.

All costs related to enteric fever were stratified by setting (tier 2/tier 3). However, nontraumatic ileal perforation costs were presented together, assuming costs would be similar across settings as it is often identified at surgery. Moreover, these ileal perforations were classified by 2 investigators (J. J. and S. K.) independently based on causality using available clinical data, laboratory evidence, and surgical or histopathological evidence. The cost for confirmed enteric ileal perforations were also presented [20]. Financial impact indicators such as distress financing and catastrophic health expenditure were computed. A household was said to be “distress financing” when they sourced their finances by borrowing (with or without interest) and selling assets [21, 22]. A household experienced catastrophic health expenditure if the household spent beyond 40% of their annual nonsubsistence expenditure on health care [23, 24]. Univariate analysis was done to explore the determinants of catastrophic health expenditure, while multivariate analysis was done to adjust for confounding.

### Ethical Considerations

The institutional review boards of the Christian Medical College, Vellore, as the coordinating institution, and all participating institutions approved the study. All patient details were collected after obtaining written informed consent from the patient/caregiver.

## RESULTS

Overall, 1275 patients provided costing information with 274 enteric fever patients from tier 2 sites, 891 from tier 3 sites, and 110 ileal perforation patients (n = 18 confirmed enteric fever perforations) from both tiers (Table 1). Of tier 2 and tier 3 patients, 31.1% and 62.9%, respectively, were younger than 15 years. More than 80% of the enteric fevers were due to *S.* Typhi in both settings. The mean duration of hospitalization was 5.6 days in tier 2, 7.95 days in tier 3, and 19.06 days among all-cause ileal perforation patients (Table 1).

**Table 1.** Demographic Characteristics of Blood Culture-Confirmed Enteric Fever Patients Included in the Study (n = 1275)

Characteristics	Tier 2	Tier 3	Ileal Perforation, All-Cause
Total	274	891	110
Organism			
Typhoid	221 (80.7)	782 (87.8)	…
Paratyphoid	53 (19.3)	109 (12.2)	…
Age, y			
0–4	28 (10.2)	228 (25.6)	22 (20)
5–14	60 (21.9)	332 (37.3)	8 (7.3)
15–29	148 (54)	243 (27.3)	34 (30.9)
30–44	33 (12)	61 (6.8)	20 (18.2)
45–59	…	…	…
≥60	5 (1.8)	27 (3)	26 (23.6)
Sex			
Male	176 (64.2)	545 (61.2)	70 (63.6)
Female	98 (35.8)	346 (38.8)	40 (36.4)
Duration of hospitalization, d, mean (95% CI)	5.6 (5.2*–*5.9)	7.95 (7.63*–*8.27)	19.06 (16.56*–*21.57)
Delay in hospitalization, d, mean (95% CI)	7.2 (6.1*–*8.3)	9.5 (8.4*–*10.6)	11.3 (7.4*–*15.1)
Annual income, Rs, mean (95% CI)	2 52 924.5 (2 22 006.5–2 83 842.4)	4 80 806.1 (4 35 837.6–5 25 774.5)	4 62 392.7 (3 49 387.7–5 75 397.8)
Annual expenditure, Rs, mean (95% CI)	1 36 775.5 (1 27 120.4–1 46 430.7)	2 43 832.6 (2 29 837.8–2 57 827.4)	187 374.8 (1 60 526.6–2 14 222.9)
Capacity to pay, Rs, mean (95% CI)	99 712.96 (90 804.2–10 8621.7)	1 88 603.3	1 35 896.3
		(1 75 087.3–2 02 119.4)	(1 10 816.8–1 60 975.8)
Health insurance, yes	43 (15.7)	202 (22.7)	2 (1.8)

Data are No. (%) except where indicated.

Abbreviation: CI, confidence interval; Rs, Indian Rupee.

The mean direct cost of enteric fever was Rs8292.3 (US$119.1) in tier 2 settings, while the same in tier 3 was Rs28 237.7 (US$405.7) (Table 2). All-cause nontraumatic ileal perforation cost was Rs84 227.5 (US$1210) while those perforations confirmed to be enteric cause (n = 18) cost Rs90 869.2 (US$1305.4). Approximately three-quarters of these costs were during the hospital stay in all patients.

**Table 2.** Distribution of Total Costs of Severe Enteric Fever

Costs	Tier 2, Rs (US$) (n = 274)		Tier 3, Rs (US$) (n = 891)	
	Mean	SE	Mean	SE
Direct medical costs				
User charges	1626.6 (23.4)	306.9 (4.4)	9995.5 (143.6)	463.0 (6.7)
Diagnostic charges	1402.2 (20.1)	114.4 (1.6)	5999.5 (86.2)	270.3 (3.9)
Drugs and consumables	3168.3 (45.5)	218.5 (3.1)	6242.0 (89.7)	496.8 (7.1)
Procedure/surgery	819.7 (11.8)	663.9 (9.5)	1279.3 (18.4)	193.5 (2.8)
Total direct medical costs	7016.7 (100.8)	1169.7 (16.8)	23 516.2 (337.8)	1087.9 (15.6)
Direct nonmedical costs				
Travel cost	336.4 (4.8)	39 (0.6)	1233.6 (17.7)	70.9 (1)
Meal cost	473.4 (6.8)	34.5 (0.5)	1347.6 (19.4)	55.4 (0.8)
Lodging charges	6.2 (0.1)	4.7 (0.1)	117.0 (1.7)	33.7 (0.5)
Informal costs	31 (0.4)	11.1 (0.2)	19.9 (0.3)	6.4 (0.1)
Other costs	428.5 (6.2)	40.1 (0.6)	685.9 (9.9)	34.1 (0.5)
Total direct nonmedical costs	1275.6 (18.3)	84.9 (1.2)	3404.1 (48.9)	128.0 (1.8)
Uncategorized others^a^	…	…	1317.4 (18.9)	687.9 (9.9)
Total direct cost	8292.3 (119.1)	1178 (16.9)	28 237.7 (405.7)	1373.4 (19.7)
Indirect cost				
Income lost by patient	1653.2 (23.8)	243 (3.5)	3415.2 (49.1)	510 (7.3)
Income lost by caretakers	2935.6 (42.2)	234.3 (3.4)	6972.7 (100.2)	565.7 (8.1)
Payment to substitute	116.9 (1.7)	58.6 (0.8)	822.9 (11.8)	321.4 (4.6)
Total indirect cost	4705.8 (67.6)	318.8 (4.6)	11 210.8 (161.1)	982.1 (14.1)

Abbreviation: Rs, Indian Rupee; SE, standard error.

^a^Uncategorized costs are costs wherein the patient/ bill carried only the total direct costs and therefore were left uncategorized.

Among the determinants of expenditure, drugs and consumables were the highest in tier 2 (38.2%), while hospital charges like consultation, bed, and administrative charges formed the highest (35.4%) in tier 3 (Table 2). Children (<15 years) with enteric fever had lower expenses than adults in both settings (Table 3). Enteric fever patients with a private hospitalization spent at least 3 times as much as patients hospitalized in a public hospital in both settings. Patients who needed intensive care (Rs54 592.7/US$784.3) spent 8 times more than regular hospitalization in tier 2 settings (Table 3).

**Table 3.** Total Direct Cost of Severe Enteric Fever by the Causative Organism, Age, Sex, and Sector

Characteristic	Tier 2		Tier 3	
	n	Mean, Rs (US$)	n	Mean, Rs (US$)
Enteric Fever	274	8292.3 (119.1)	891	28 237.7 (405.7)
Organism				
* Salmonella* Typhi	221	9061.9 (130.2)	782	28 337.3 (407.1)
* Salmonella* Paratyphi	53	5083.7 (73)	109	27 523.3 (395.4)
Age, y				
<15	88	6975.7 (100.2)	560	18 396.2 (264.3)
≥ 15	186	8915.3(128.1)	331	44 888.1 (644.9)
Sector				
Public sector	133	4308.8 (61.9)	331	9205.9 (132.3)
Private/charity	141	12 049.8 (173.1)	560	39 486.9 (567.3)
Sex				
Male	176	7069.5 (101.6)	545	28 335.4 (407.1)
Female	98	10 488.5 (150.7)	346	28 083.8 (403.5)
ICU admission				
No	264	6538.5 (93.9)	823	25 169.9 (361.6)
Yes	10	54592.7 (784.3)	68	65 367.2 (939.1)
Outcome				
Recovered without complications	247	7008 (100.7)	857	28 043.9 (402.9)
Sequelae/death/referred	23	21 722.9 (312.1)	12	68 443.3 (983.3)
Duration of stay, d				
≤3	56	4582.8 (65.8)	48	15 170.2 (217.9)
3–7	168	7086.1 (101.8)	492	24 996.6 (359.1)
>7	50	16 500 (237)	351	34 567.8 (496.6)
Insurance				
No	231	8270.4 (118.8)	689	27 132.4 (389.8)
Yes	43	8410.1 (120.8)	202	32 007.7 (459.8)

Abbreviation: ICU, intensive care unit; Rs, Indian Rupee.

The average productive time lost by the patient and caregivers together in different activities was 207 hours in tier 2, 263.5 hours in tier 3, and 535 hours for ileal perforations confirmed to be due to enteric fever. The indirect cost of enteric fever was Rs4705.8 (US$67.6) among patients in tier 2, while it was Rs11 210.8 (US$161.1) in tier 3 settings (Table 2). The indirect cost of ileal perforation of any cause was Rs26 488.2 (US$380.5) while the same among ileal perforations confirmed to be due to enteric fever was Rs46 770 (US$671.9).

**Figure 1.**
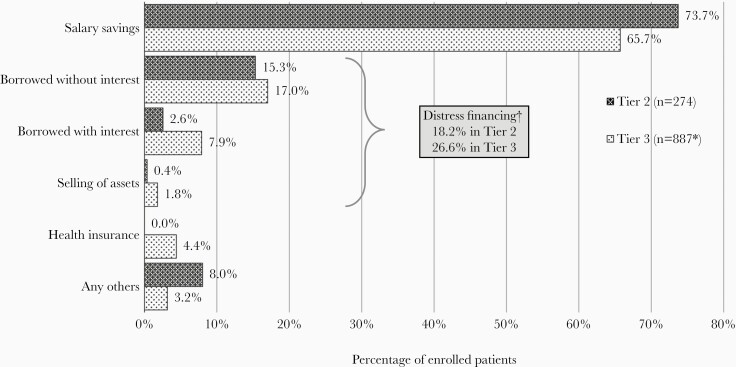
Source of financing for severe enteric fever. Tier 2; surveillance in smaller hospitals in 5 rural sites and 1 urban site; tier 3, surveillance in 8 key tertiary care hospitals. *Four patients in tier 3 did not report their source of finance. †A person is said to be under distress financing when they spend by borrowing or selling of assets.

Patients with enteric fever predominantly used their salary or savings (73.7% and 65.7% in tier 2 and tier 3, respectively) to finance their treatment costs (Figure 1). No patients in tier 2 settings utilized health insurance, although 15.7% (n = 43) reported having some form of health insurance. In tier 3, 4.4% availed health insurance, although 22.7% (n = 202) reported having health insurance coverage. In tier 2 and tier 3 settings, 18.2% (n = 50) and 26.6% (n = 237), respectively, of the enteric fever patients were forced to distress finance their expenses (Figure 1). The costs for all-cause nontraumatic ileal perforation forced 39% of the households into distress financing their expenses while the costs due to confirmed enteric perforations forced 66.7% of the households into distress financing.

**Figure 2.**
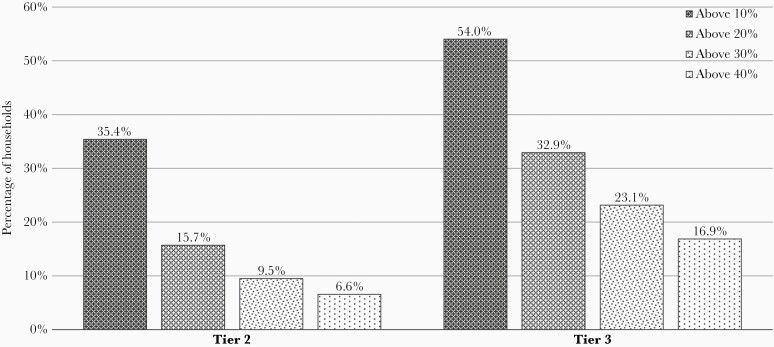
Percentage of households spending above the cutoff on health care due to enteric fever. Distribution of direct medical expenses for enteric fever as a proportion of capacity to pay.. Catastrophic health expenditure was not computed for 1 subject with no expenses.

In tier 2, 35.4% (n = 97) of households spent over one-tenth of their nonsubsistence expenses on enteric fever treatment, while it was 54% (n = 481) of the households in tier 3 for the same (Figure 2). In tier 2 and tier 3 settings, 6.6% (n = 18) and 16.9% (n = 150), respectively, experienced catastrophic health expenditure (Figure 2). With regard to ileal perforations, 49.1% of the households with all-cause perforations and 61.1% of households with confirmed enteric fever as the cause experienced catastrophic health expenditure.

Multivariate analysis showed that, in both settings, hospitalization in the private sector compared to the public sector (tier 2, adjusted odds ratio [aOR], 12; 95% confidence interval [CI], 1.5–95.2: tier 3, aOR, 2.7; 95% CI, 1.7–4.3) and lowest income quintile compared to higher-income quintiles (tier 2, aOR, 7.0; 95% CI, 2.3–21.5: tier 3, aOR, 5.3; 95% CI, 3.5–8.2) had higher odds of experiencing financial catastrophe. Furthermore, families with lower than primary education (aOR, 7.6; 95% CI, 2.1–27.6) in tier 2 and patients older than 15 years (aOR, 2.9; 95% CI, 1.9–4.3) in tier 3 had a higher risk of catastrophic health expenditure compared to their counterparts (Table 4).

**Table 4.** Determinants of Catastrophic Health Expenditure

Determinant	Tier 2 (n = 274)				Tier 3 (n = 890^a^)			
	n	%	Adjusted Odds Ratio	*P* Value	n	%	Adjusted Odds Ratio	*P* Value
Total	18	6.6			150	16.8		
Highest education of the household								
Less than Primary	6	28.6	7.56 (95% CI 2.07*–*27.57)	.002	Education details not collected in this surveillance			
More than Primary	12	4.7	Ref					
Household size								
Up to 3 members	2	3.8			40	22.1	1.33 (95% CI 0.86*–*2.06)	.203
More than 3 members	16	7.2			110	15.5	Ref	
Sector								
Public	1	0.8	Ref	.019	30	9.1	Ref	<.0001
Private/Charity	17	12.1	12.02 (95% C 1.52*–*95.19)		120	21.5	2.69 (95% CI 1.69*–*4.28)	
Age, y								
<15	7	8.0			65	11.6	Ref	<.0001
≥15	11	5.9			85	25.8	2.87 (95% CI 1.91*–*4.3)	
Sex								
Male	10	5.7			97	17.8		
Female	8	8.2			53	15.3		
Insurance								
No	14	6.1			118	17.2		
Yes	4	9.3			32	15.8		
Income quintile								
Q1, poorest	12	18.5	6.95 (95% CI 2.25*–*21.52)	.001	60	33.3	5.31 (95% CI 3.45*–*8.17)	<.0001
Q2 and above	6	2.87	Ref		90	12.7	Ref	

Univariate logistic regression was used to find early predictors and only those found to have an association was built into the regression model to identify predictors.

^a^Catastrophic health expenditure could not be computed for 1 patient in tier 3 as he/she did not report any expenses.

## Discussion

Overall, the direct cost of enteric fever requiring hospitalization was estimated to be US$119.1 in tier 2 surveillance and US$405.7 in tier 3 surveillance, which was higher than the DOMI study (2003) estimates from Kolkata, India (US$29) [11]. Similarly, the direct costs in tier 3 settings were higher than the total cost of typhoid fever requiring in-patient care from the Delhi study (1995–1996) done in urban slums. It is noted here that the total costs in the Delhi study included the patient costs and the cost borne by the health system [13]. Both these previous studies were >2 decades old, and health care costs are likely to have changed over this time.

Studies from the Surveillance for Enteric Fever in Asia Project (SEAP 2016–2018) from Pakistan, Nepal, and Bangladesh (Ranged between US$ 168.72 - 316.94) done in teaching hospitals, reported slightly lower directs costs among inpatients and indirect costs comparable to our estimates from similar tier 3 surveillance (direct cost US$405.7; indirect cost US$161.1) [25–27]. These studies excluded medical costs that were not related to enteric fever. In our study all costs related to the Acute Febrile Illness were captured. Also, the use of telephonic data collection could run the risk of underestimating the costs in these studies.

The cost of nontraumatic ileal perforation due to enteric fever has not been previously studied in India. Unsurprisingly this complication of enteric fever led to approximately 3 times the costs of a noncomplicated enteric fever in our study. This shows the direct costs for a hospitalized case of enteric fever could range from US$119.1 to US$1305.4, depending on the setting and severity.

None of the tier 2 and only 4.4% (n = 39) of tier 3 patients could access health insurance for their treatment costs. The Rashtriya Swasthya Bima Yojana (RSBY) and Ayushman Bharath (AB-PMJAY-2018) are national schemes aimed to enable the “poorest 40 percent of the population to meet the expenses for quality secondary and tertiary care. Thereby it was aimed to reduce catastrophic expenditure and remove the financial risk arising out of such episodes” [28–31]. However, the study participants could not access these schemes and had to pay for care largely out of pocket. Consequently, 45% (n = 287) of enteric fever households resorted to distress financing. The lowest income quintile group in both the tiers (18.5% and 33.3%) had the highest proportion of catastrophic spending (Table 4). Therefore, national health insurance schemes need to expand coverage and enroll a wider network of health care providers to protect the poor. Universal health insurance schemes and universal basic income approaches could pave the way for equitable access to health care and also reduce impoverishment in the country.

Patients who sought care in the private sector had 3 times greater expenditure compared to the public sector and therefore had higher odds (aOR, 12.0 and 2.7 for tier 2 and tier 3, respectively) of experiencing catastrophic health expenditure. The cost of treatment for enteric fever requiring hospitalization in tier 3 (US$405.7) was higher than the cost of dengue hospitalization (US$197.03) in teaching hospitals in India [32]. This shows that enteric fever treatment is expensive and results in high out-of-pocket payments, similar to ailments due to other infectious causes.

The data collected through the SEFI study shows that out-of-pocket patient expenditure for the treatment of enteric fever in India remains high in both tiers. The disease continues to disproportionately coerce households into financial catastrophe. To prevent this, control measures against the disease, such as the typhoid conjugate vaccines, must be deployed after appropriate cost-effectiveness studies.

Our study is the first large study to report patient-level costs of enteric fever requiring hospitalization in India, studying costs from 1275 patients from 14 sites. Previous studies in India were from 1 or 2 sites, and the costs were derived from a significantly lower study sample [11, 13].

There were limitations to the study. Firstly, while we attempted to represent the costs in different risk settings, we recognize the limitation in being truly representative of the country. This study does not provide adequate information on the costs related to enteric fever in very poor urban slums where most patients tend to get treated in informal care as outpatients with prophylactic antibiotics. Secondly, we recognize that a variable proportion of health care costs are absorbed by the health system in both the government and private charitable facilities, and a comprehensive economic burden requires estimation of the health system costs. However, collecting costs from health care providers require multiple levels of approval, particularly in the government sector. Therefore, these costs were not computed and our focus remained predominantly on out-of-pocket expenditure. Further, consumption expenditure details obtained in our study might be affected by recall bias as participants were required to provide their last month’s complete expense details. Also, we assumed an 8-hour schedule of work per day for computation of indirect costs and did not collect actual work timings. We did not monetize wages for children, unemployed, and homemakers, which could underestimate the indirect cost of the disease.

## CONCLUSION

This study shows that out-of-pocket expenses for enteric fever are high in urban areas and for hospitalizations. Patients and their households bear high indirect costs, particularly detrimental for those from lower strata who access public services intended to provide free health care. The study shows that enteric fever continues to push families into impoverishment and that the government health insurance schemes for the poor have not proven very effective in averting their financial risk. It is hoped that these results will help build a case for developing and implementing effective control measures to minimize the effects it bears on enteric fever patients due to out-of-pocket expenditures.

## Supplementary Data

Supplementary materials are available at *The Journal of Infectious Diseases* online. Consisting of data provided by the authors to benefit the reader, the posted materials are not copyedited and are the sole responsibility of the authors, so questions or comments should be addressed to the corresponding author.

## Notes

***Acknowledgments.*** We acknowledge with gratitude the participants and their families for their partnership over the 2 years of surveillance. We acknowledge Miriam Thankam George, Vijay Anand Ismavel, Anna P. Alexander, Dennis Martin, Pradeep Zacharia, Balaji Veeraraghavan, Anuradha De, Sachee Agrawal, and Deepak S. Singh for their support at various stages of the surveillance. In addition to members of the Surveillance for Enteric Fever consortium, we acknowledge Jason Andrews, Nathan Lo, Gordon Dougan, Robert Breiman, Megan Carey, and Thomas Cherian for their contribution to the design and analytic plans. Supriya Kumar, Duncan Steele, and Anita Zaidi of the Bill and Melinda Gates Foundation have been steadfast in their support of the study with constructive comments and encouragement. Our gratitude is also due to Soumya Swaminathan, former Secretary, Department of Health Research, Government of India, for providing leadership and participation from the Government in the design and conduct of the surveillance.

***Author contributions.*** J. J., G. K., and V. R. conceived and designed the study. J. J., G. K., D. K., S. P., A. S. C., A. S. K., K. R., P. S. P., and V. R. developed the study protocol and analysis plan. J. J. and G. K. coordinated the project. R. R., S. K., D. K., K. R., A. S. K., S. E. E., M. S. E., M. G., A. S. H., D. R. J., S. T., R. M. K., C. D. S., A. K., J. S., K. S., S. P. P., S. N., S. A., M. T., and P. B. coordinated data collection at the sites. D. K., P. S. P., A. S., S. K. R., and S. P. analyzed the data. D. K., A. S., and S. K. R. drafted the manuscript. All authors have reviewed the manuscript and approved it. J. J. and G. K. had complete access to data and guaranteed the manuscript.

***Disclaimer***. The funder had no role in the design analysis or interpretation of the findings.

***Financial support.*** This work was supported by Bill and Melinda Gates Foundation (grant number INV-009497-OPP1159351) as part of the National Surveillance System for Enteric Fever in India.

***Supplement sponsorship.*** This supplement is sponsored by the Christian Medical College Vellore Association.

***Potential conflicts of interest.*** All authors: No reported conflicts of interest. All authors have submitted the ICMJE Form for Disclosure of Potential Conflicts of Interest. Conflicts that the editors consider relevant to the content of the manuscript have been disclosed.
